# Lunar cycle and moonlight intensity influence nocturnal migration patterns in a small songbird

**DOI:** 10.1038/s41598-025-04270-3

**Published:** 2025-06-06

**Authors:** Dajana Prinz, Ramona Julia Heim, Moritz Meinken, Nick Niemann, Laurin Temme, Alexandra Esther, Wieland Heim

**Affiliations:** 1 Institute of Landscape Ecology, University of Muenster, Muenster, Germany; 2 City of Emsdetten, Urban Development and Environment, Emsdetten, Germany; 3 Department of Evolutionary Biology and Environmental Studies, University of Zurich, Zurich, Switzerland; 4 Vertebrate Research, Federal Research Centre for Cultivated Plants, Institute for Plant Protection in Horticulture and Urban Green, Julius Kuehn Institute, Braunschweig, Germany; 5 Present Address: Institute for Biology and Environmental Sciences, University of Oldenburg, Oldenburg, Germany

**Keywords:** Light, Lunar cycle, Migration, Moon, Navigation, Passerine, Animal migration, Animal behaviour

## Abstract

Lunar cycle and moonlight exposure have significant impacts on animal behaviour and physiology. The presence or absence of moonlight, along with predictable changes in brightness throughout the lunar cycle, can shape reproduction, foraging, communication, and other aspects of an animal’s world. While it has been shown that invertebrates use the moonlight for orientation, little is known on the effect of the lunar cycle on migratory birds. We found that the lunar cycle affected the nocturnal migration activity of a diurnal songbird species, the Eurasian Skylark *Alauda arvensis.* The occurrence of birds increased with moon fraction, moonlight intensity and duration, while abundance correlated positively with increasing moonlight intensity. Our findings of increased migration activity in bright nights around full moon contradict previous assumptions that small bird migrants would avoid such nights due to increased predation pressure and decreased visibility of stars for orientation. We argue that migrants relying on visual cues for orientation might favour moonlit nights, while future studies should also test whether the position of the moon can be used for navigation by birds.

## Introduction

The lunar cycle and moonlight exposure have significant impacts on animal behaviour and physiology^1–5^. Most effects are linked to the predictable changes in brightness across the lunar cycle, with different species preferring lighter or darker nights^4,6^. The presence or absence of moonlight can shape reproduction, foraging, communication, and other aspects of an animal’s world on land or under water^1,3,4,7–9^.

However, little is known regarding the effects of the moon on animal migration in general. It was shown that the vertical migration of zooplankton during the Arctic winter is following the lunar cycle^1^, and that migratory patterns in shrimp and micronekton are also correlated with the lunar phase^10,11^. The moon is furthermore used as a compass by flying insects during migration^12^, and more insects were found to migrate during moonlit nights in a recent radar study^13^.

Even less is known regarding the effects of the lunar cycle on the migration of birds. Interestingly, a technique called “moon watching” became popular to survey the intensity of bird migration starting in the 1950´s^14,15^. However, these studies demonstrated only that nocturnally migrating birds become visible against the bright moon (while they cannot be seen by humans against a dark sky), but it remained unknown whether the lunar cycle would affect the number of migrants.

The moon could favour the migration of birds by being a compass or a landmark and/or by making landmarks better visible^16,17^. However, moonlight might also hinder bird migration because of increased predation risk by nocturnal predators, such as large owls^18^. Furthermore, bright moonlight could distract the star-based orientation which many migratory birds rely on^16^. Depending on whether a species only migrates at night or also during the day, which food source it prefers, and whether it is large or small, experienced, or unexperienced, the moon-related mechanisms that determine migration could differ. For example, a negative effect of lunar illumination on the number of captured migrants was found in a small nocturnal owl species, possibly due to higher predation risk^19^. In contrast, other nocturnal migrants such as nightjars showed increased migration activity after full moon periods in tracking studies^20,21^, which could be attributed to better re-fuelling possibilities for these visually guided species in bright nights due to more time available for foraging.

While most of our knowledge on the effects of the moon on bird migration stems from studies of larger, nocturnal species, very few studies have investigated the effects of the lunar cycle on diurnal species that only migrate at night, such as many of the small songbird species. For example, research on caged Savannah Sparrows *Passerculus sandvicensis* did not find evidence of the moon serving as a compass or landmark^17^. In another experiment, *Zonotrichia* sparrows were found to be affected by moonlight—while orientation was found to be hampered under moonlit conditions, migration activity increased^22^. Increased migration activity was also described for free-flying Eurasian Skylarks *Alauda arvensis* during the moon´s waxing phase, and the authors suggested that these birds selected bright nights that facilitated navigation by increased visibility of topographic cues^23^.

Overall, the impact of the moon on bird migration has been largely overlooked, with current reviews often completely disregarding its role in orientation and navigation^24^. The exact navigation methods used by birds during migration remain unknown^25,26^. Therefore, gaining an understanding of the moon’s effects on bird migration could be a significant step towards answering this question.

Proxies for night-time brightness, such as moon fraction and moonlight intensity, are thought to impact foraging efficiency and predator avoidance, potentially affecting birds’ energy reserves and migration decisions^21^. Additionally, the timing of moonrise and moonset may provide temporal cues for migration initiation or intensity^27^. The duration of moonlight throughout the night could also play a role in extending periods of increased activity^20^. Understanding these diverse lunar influences is crucial for comprehending the complex interplay between celestial conditions and avian migration patterns.

We selected the Eurasian Skylark (hereafter: skylark) as a model species for two reasons. First, this otherwise strictly diurnal songbird is one of few species known to migrate regularly during both day and night^28^, and might have therefore evolved a range of orientational/navigational capabilities. Second, there is prior evidence that skylark migration activity could be linked to the lunar cycle, with highest numbers observed during the moon´s waxing phase^23^.

We anticipate that the effects of the moonlight on the migration activity of this small songbird may exhibit a multifaceted nature. While increased moonlight could potentially have a negative impact by elevating predation risk, it is also plausible that the moon serves as a navigational aid, either as a compass or by enhancing the visibility of ground landmarks. Therefore, this study aims to empirically assess the influence of moon phase, moon rise and moonlight duration on the migration of a small landbird, recognizing the potential for both inhibitory and facilitative effects, and ultimately contributing to a more comprehensive understanding of avian migration.

## Results

**Fig. 1:**
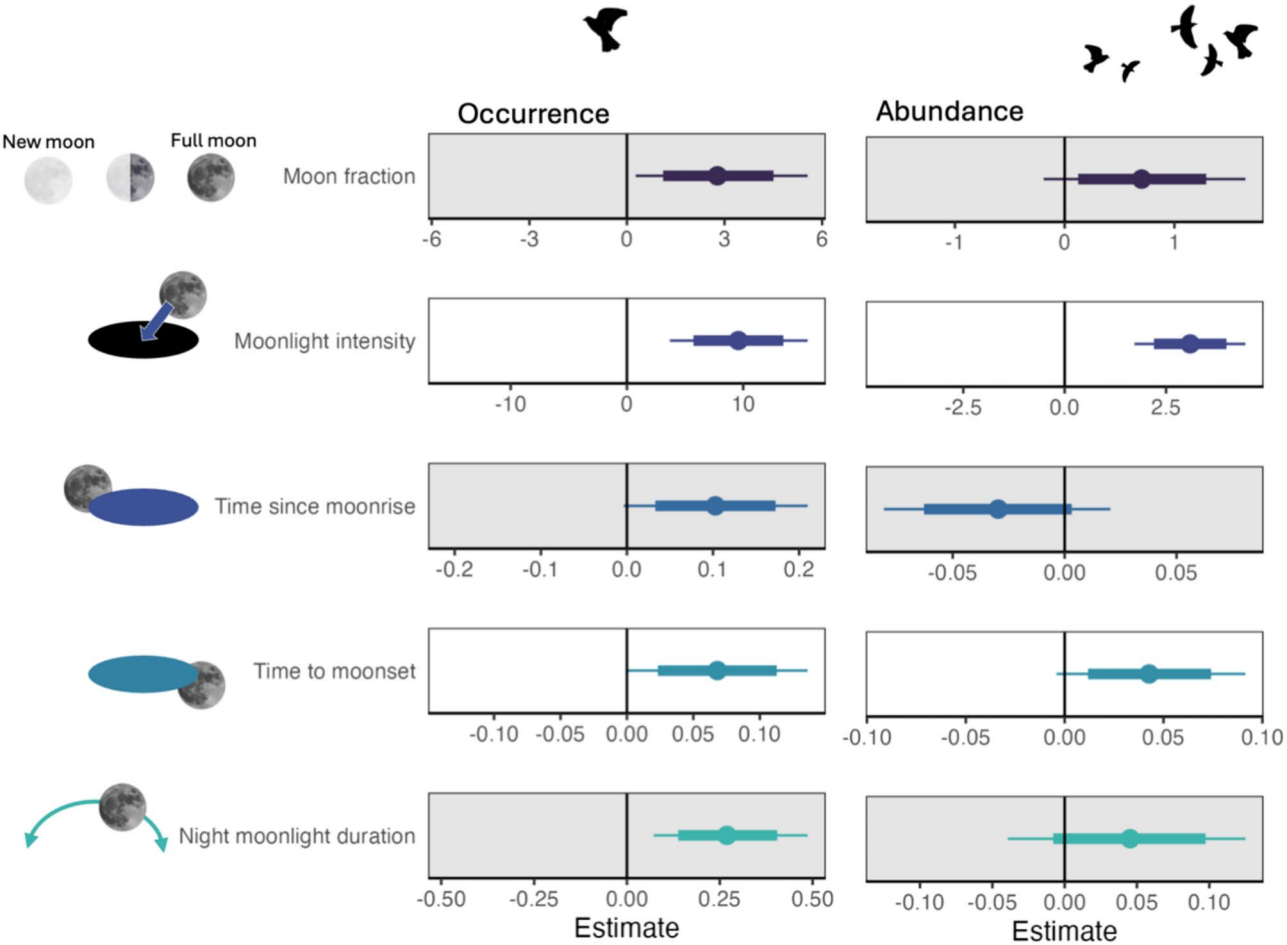
Model coefficients of five moon variables (moon fraction, moonlight intensity, time since moonrise, time to moonset, and moonlight duration per night). For each variable, two models are presented: one with skylark occurrence per hour (binomial), and another with skylark abundance per hour (Poisson: number of caught skylarks, excluding zeros) as the dependent variables. Colored dots represent means, thin lines show 95% credible intervals (CrIs), and thick lines indicate 80% CrIs. Effects are considered significant when the 95% CrI does not cross the black zero line. See methods section for further details.

Our analysis revealed that moonlight significantly influenced both occurrence (defined as the likelihood of catching skylarks during migration), and abundance (defined as the number of skylarks caught; Fig. 1, Supplementary Table S4). Moon fraction, representing the portion of the moon’s visible disk illuminated by sunlight (increasing towards full moon), showed a positive significant effect on skylark occurrence (mean: 2.78, 95% CrI: 0.27, 5.56). While the effect on abundance was also positive, it was not significant (mean: 0.70, 95% CrI: − 0.19, 1.65). Moonlight intensity demonstrated strong positive effects on both occurrence (mean: 9.59, 95% CrI: 3.69, 15.53) and abundance (mean: 3.10, 95% CrI: 1.72, 4.46) of skylarks, indicating a substantial increase in both the likelihood of catching skylarks and their numbers under brighter moonlight conditions (Fig. 1).

The temporal aspects of moonlight showed less pronounced effects (Fig. 1). Time since moonrise had no significant impact on either occurrence (mean: 0.10, 95% CrI: 0.00, 0.21) or abundance (mean: − 0.03, 95% CrI: − 0.08, 0.02) of migrating skylarks. Similarly, time to moonset showed no significant effects on occurrence (mean: 0.07, 95% CrI: 0.00, 0.14) or abundance (mean: 0.04, 95% CrI: 0.00, 0.09). Night moonlight duration positively affected skylark occurrence (mean: 0.27, 95% CrI: 0.07, − 0.49), but had no significant impact on abundance (mean: 0.05, 95% CrI: − 0.04, 0.13). Generally, we found no evidence for interannual variation; the year variable did not significantly affect skylark occurrence or abundance in any of our models.

## Discussion

We showed that multiple aspects of the lunar cycle significantly correlate with the migration activity of a small songbird. We found the highest probability of capturing migrating skylarks (occurrence) in nights closest to the full moon and/or with the highest moonlight intensity and/or moonlight duration, while the number of captured birds (abundance) increased with moonlight intensity. Our results are coherent with earlier observations of increased migration activity in skylarks closer to full moon^23^.

Most studies that found an effect of the lunar cycle on the movements of birds have explained their observations with the differences in illumination. For example, the increased flight height of swifts during moonlit nights has been attributed either to nocturnal foraging or to avoidance of nocturnal predators^7,29^, while the link between full moon and migration phenology in lunarphilic nightjars has been explained with the tracking of prey availability^21^. More light at night is known to increase the activity of nocturnal predators^20,30,31^. If nocturnal predation pressure would be increased in moonlit nights, we would expect lower numbers of migrating skylarks during full moon like in other small bird migrants^19,32,33^, and not the highest as observed by us and an earlier study on the same species^23^.

In contrast to this, better conditions for the usage of visual cues could be an explanation for the increased migration activity of skylarks in moonlit nights. In seabirds, it has been shown that arrival times were synchronized with the lunar cycle^34^, and that longer distances are travelled in moonlit nights^35,36^, with the latter mostly linked to better conditions for visual foraging. Diurnal species, such as skylarks, likely generally rely more on visual cues, including during migration phases, and might therefore prefer bright, full-moon nights for nocturnal flight bouts. Nocturnal activity of otherwise diurnal species during full-moon nights has been reported from other species as well, e.g. Northern Lapwings *Vanellus vanellus*^37^.

We found that moonlight duration per night had a significant positive effect on the occurrence of skylarks, while we observed no effect on abundance. Skylarks might favour nights for migration with more constant illumination, rather than migrating during nights with only a limited number of bright hours.

Furthermore, we observed a tendency of increasing skylark occurrence with time since moonrise and time to moonset, hinting at higher migration activity during times when the moon is high in the sky. The higher the moon ascends in the sky, the more illuminated is the surrounding landscape, which could again facilitate migration following visual cues such as topographic features^17^.

Alternatively, birds might use the position of the moon for navigation directly. So far, this has been considered unlikely, given the periodic and quickly changing appearance of the moon^24^. However, we consider it possible that if birds can interpret the changing position of the sun, they might also be able to understand the changing but predictable pattern of the lunar cycle, and use it for determining direction. Evidence for departure and arrival timings synchronized with the lunar cycle underpin the possibility that birds use the position of the moon as navigational aid^34,38^. Further evidence comes from studies on invertebrates^39^, which are known to use the polarization of moonlight for orientation^40^, and for which an inborn moon compass has been confirmed^41^.

Our overall argumentation assumes that the number of migrating skylarks aloft correlates with the number of trapped individuals on the ground. However, we cannot rule out the possibility that skylarks were misoriented in moonlit nights^22^ and were therefore more likely to be trapped in our nets. However, given that the nets used for trapping are much more visible in nights with moonlight, we would still expect a lower number of trapped individuals around full moon. Alternatively, migrating birds may alter their flight behaviour in connection with the lunar cycle. A recent study found that the nocturnal flight call rate increases with lunar illumination, which the authors interpreted as a tendency towards lower flight altitudes in brighter nights^42^. However, higher flight altitudes were found for swifts in moonlit nights^7,29^. We cannot rule out that flight altitude could affect the capture probability, because lower-flying birds could have a higher chance to hear the playback used in our study.

In summary, we call for more attention towards the over-looked importance of the lunar cycle in migration studies, especially regarding migratory species relying on visual cues (e.g. landmarks instead of magnetoreception) for orientation, which might prefer moonlit nights. Future studies should evaluate whether diurnal species that migrate almost exclusively at night also show a preference for moonlit nights, or whether the pattern observed in skylarks is only found in species with a general preference for diurnal migration. Further studies using radar, which can identify the number of actively migrating individuals in the sky^43^, in combination with moon-watching sensors and acoustic recording units^44^ would be desirable to provide further evidence for lunar cycle driven migration activity^42^. On top of that, future studies should also test whether the position of the moon can be used for navigation by birds.

## Methods

### Study species

The Eurasian Skylark is a widely distributed breeding bird species in the Palearctic with about 44,3 million-78,8 million pairs in Europe^45^. The habitats of skylarks are open agricultural landscapes that are heavily impacted by agricultural intensification and land abandonment^46,47^, leading to significant gaps in their distribution and a global decline in population^45,48^. During migration, skylarks are exposed to hunting^49^, and possibly affected by pesticides^50^. In autumn, most of the Western Palearctic skylarks migrate from their breeding area to south-west Europe, to the Mediterranean region or to the Near East and up to the northern edge of the Sahara, whereas southern populations often are residents^51^. Skylarks migrate both during day and at night^28^, although they are generally a diurnal species. They cover distances of up to 250 km in one flight bout^28^ and tend to migrate without forming close flocks but stay in contact through migration calls^52^.

### Fieldwork

We conducted fieldwork in a small (50 × 80 m) maize stubble field at the Julius-Kühn-Institute, Federal Research Centre for Cultivated Plants, northwest of the city of Münster, Germany (51.97548°N, 7.56540°E). The breeding density of skylarks in Münster is extremely low^53^ and it can be assumed that the caught individuals are not local birds but migrants.

Migrating skylarks (n = 1533 individuals) were trapped during autumn (n = 56 nights) over a period of three years (2018–2020). We used four vertical mist-nets between September and November in 2018 (twice 18 × 3 m with 19 mm mesh size and twice 18 × 3 m with 30 mm mesh size) and six mist-nets in 2019 and 2020 (four nets 18 × 3 m with 19 mm mesh size and twice 18 × 3 m with 30 mm mesh size). Parallelly arranged mist-nets were placed with two nets in a row and positioned across the main migratory direction, which is assumed to be southwest in autumn in Germany^51^. We placed a tape lure with the calls and song of skylark in the middle of the mist-nets. It can be assumed that birds were attracted solely by the tape lure, as no skylarks were observed when no playback was used. We opened nets at the earliest one hour before sunset and closed them at the latest one hour after sunrise. They were controlled one time per hour. Fieldwork was interrupted and cancelled respectively during strong wind, heavy rain and if the nets were frozen.

All required permits for the capture and ringing of birds were issued by the Ministerium für Umwelt, Landwirtschaft, Natur- und Verbraucherschutz of North-Rhine Westfalia and the bird ringing centre at the Institute of Avian Research, Wilhelmshaven. All experiments were performed in accordance with relevant named guidelines and regulations. No animals were harmed during the study.

### Astronomical and weather data

We selected moon fraction, moonlight intensity, time since moonrise, time to moonset, and night moonlight duration as our lunar variables to comprehensively capture various aspects of moonlight conditions. These variables collectively represent the moon’s visibility, light intensity, temporal positioning, and duration of influence, allowing us to thoroughly examine the effects of moonlight on night-time skylark migration numbers. Moon fraction, representing the portion of the moon’s visible disk illuminated by sunlight (ranging from 0.0 for new moon to 1.0 for full moon), was derived using the *suncalc* package in R. This value indicates how much of the moon’s surface appears lit from the Earth’s perspective. Moonlight intensity, the predicted lunar illumination relative to an “average” full moon, was calculated using the *moonlit* package. This measurement takes into account a comprehensive set of factors including geographical location, date and time, lunar position and phase, celestial distances, atmospheric conditions, and twilight effects. Moonrise and moonset times for specific dates were obtained using the *suncalc* package in R. To calculate the time since moonrise, we determined the duration between the moonrise time and the time of our skylark observation (= time when the nets were checked). Similarly, time to moonset was computed as the duration from the observation time to the upcoming moonset. Night moonlight duration, representing the hours between moonrise and moonset minus hours with moon after sunrise or before sunset, was calculated using astronomical data downloaded from https://www.sunrise-and-sunset.com and https://www.timeanddate.de (accessed 30 March 2021).

The meteorological station of the Institute of Landscape Ecology, University of Münster, provided weather data comprising cloud cover (estimated in eight categories from 0 = no clouds to 8 = completely covered), maximum wind speed [m s^−1^], temperature [°C], and visibility [km] for our study. The data was recorded for ten minutes in each case. To get meteorological data for every mist-netting hour, the last six values before this hour were averaged (for cloud cover, temperature and visibility) or the maximum value was used (maximum wind speed). We excluded precipitation from our analysis due to its rarity in our dataset, with only four observations recording rainfall, all of which coincided with zero skylark captures. For the distribution of the data see Supplementary Figures S1 and S3.

### Data analysis

To analyse the impact of the moon on skylark migration, we run linear mixed effects models with R version 4.3.0^54^ in a Bayesian framework using the R-INLA package^55^. The INLA (Integrated Nested Laplace Approximation) algorithm is an analytic approximation using the Laplace method, which is less computationally intensive and thus faster than the simulation-based Monte Carlo integration^56^. We used INLA default priors, which are flat priors.

The moon variables (moon fraction, moonlight intensity, time since moonrise, time to moonset, night moonlight duration) were included as fixed effects together with the year (factor levels: 2018, 2019, 2020), to control for potential effects of the different sampling years. We fitted separate models for each moon variable as the moon variables were closely linked (see Supplementary Figure S2). For each moon variable we fitted two separate models: a binomial model to assess the effects of moon variables on the occurrence of migrating skylarks (presence/absence) and a Poisson model to evaluate their effects on abundance. This approach allowed us to disentangle the variables influencing these two distinct aspects of skylark migration. Modeling occurrence and abundance separately also avoided the complexity of zero-inflated models, which can obscure results by combining these processes into a single framework, potentially leading to difficulties in interpretation and overfitting due to the combined estimation of true zeros and abundance. For the first model, we used a variable that indicated if there were skylarks caught during this observation (1) or not (0). We therefore fit the models with family = “binomial”. To understand the effects of the moon on skylark abundance, we only used observations in which skylarks were caught and included the number of birds caught as dependent variable and used family = “poisson”. For the models of time since moonrise and time to moonset, we refined our dataset to include only observations when the moon was present in the sky. This restriction was crucial, as calculating the time since moonrise or time until moonset is only meaningful for skylark migration when the moon is actually visible. Our primary interest was to investigate the effects of lunar conditions on skylark migration. To isolate these lunar effects and control for other environmental and temporal factors that might influence skylark migration, we furthermore included cloud cover (numeric, model = “rw1”), visibility (numeric, model = “rw1”), max wind speed (numeric, model = “rw2”), temperature (numeric, model = “rw2”), julian date (day of the year, numeric, model = “rw2”), and night (factor, model = “iid”). In INLA, “rw1” refers to a first-order random walk model, which assumes smooth, gradual changes between adjacent values. The “rw2” is a second-order random walk, allowing for more flexibility in modeling non-linear relationships. The “iid” model assumes independent and identically distributed random effects, suitable for categorical variables like night.

## Supplementary Information

Supplementary Information.

## Data Availability

All data and R codes were uploaded on the DARE research repository of the University of Oldenburg (https://dare.uol.de) and are publicly available (DOI: 10.57782/76HP38).
