# Supplementary material for: Lunar cycle and moonlight intensity influence nocturnal migration patterns in a small songbird
Source: Sci Rep. 2025 Jun 6;15:19944. doi: 10.1038/s41598-025-04270-3 (PMC12144127; doi:10.1038/s41598-025-04270-3)
Supplement: Supplementary file 1 — Supplementary Information. [file 41598_2025_4270_MOESM1_ESM.docx]

**Supplementary information to**

**Lunar cycle and moonlight intensity influence nocturnal migration patterns in a small songbird**

Dajana Prinz^1,2^, Ramona Julia Heim^1,3^, Moritz Meinken^1^, Nick Niemann^1^, Laurin Temme^1^, Alexandra Esther^4^, Wieland Heim^1,3,5*^

^1^Institute of Landscape Ecology, University of Muenster, Muenster, Germany

^2^City of Emsdetten, Urban development and environment, Emsdetten, Germany

^3^Department of Evolutionary Biology and Environmental Studies, University of Zurich, Switzerland

^4^Julius Kuehn Institute, Federal Research Centre for Cultivated Plants, Institute for Plant Protection in Horticulture and Urban Green, Vertebrate research, Braunschweig, Germany

^5^Present address: Institute for Biology and Environmental Sciences, University of Oldenburg, Germany

DP and RJH contributed equally.

*Corresponding author: Wieland Heim Email: wieland.heim@uol.de

**
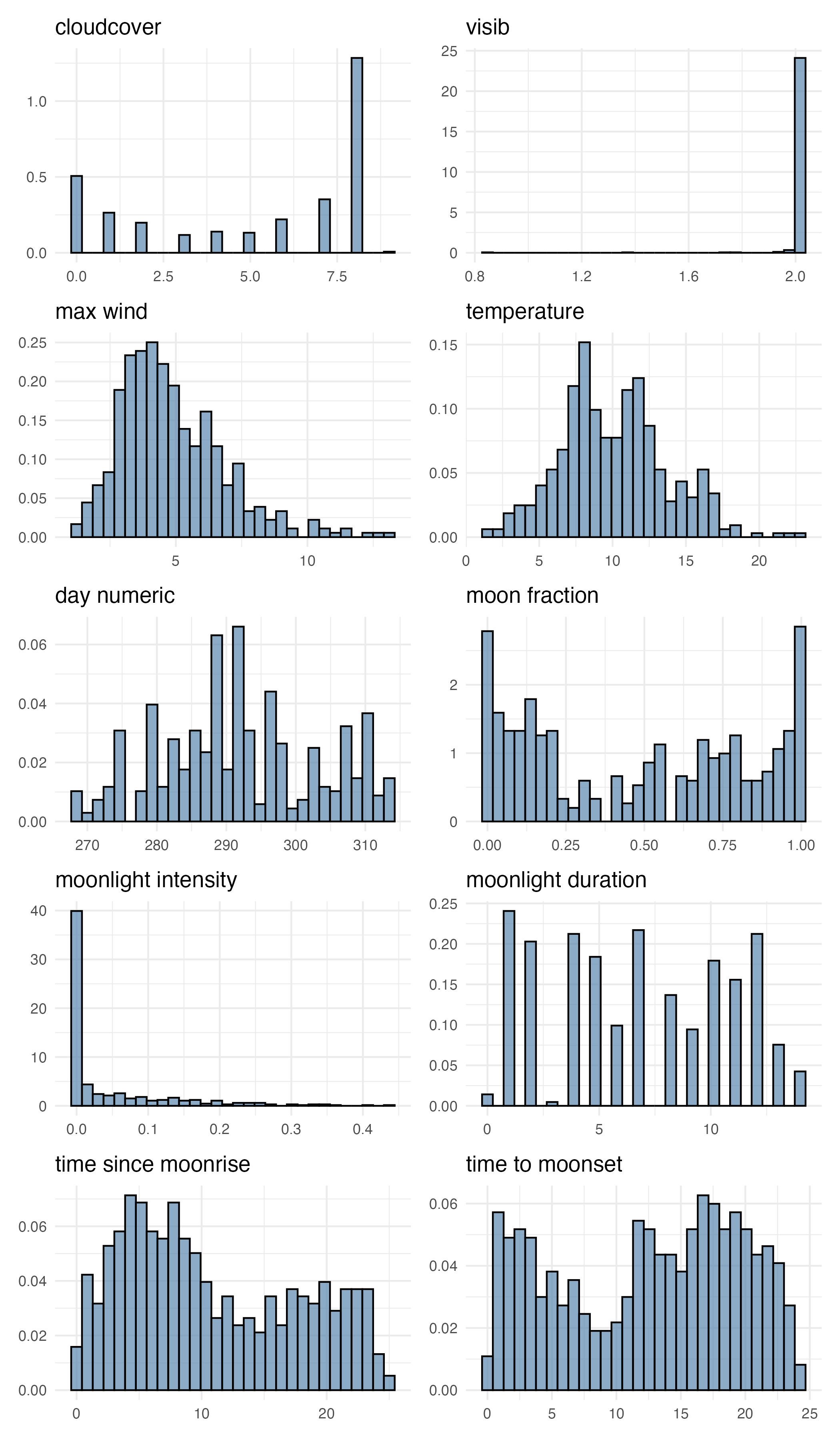
**

**Supplementary Figure S1**: *Distribution of continuous variables used in the models displayed as histograms. The x-axis shows the observed values of the respective variable, while the y-axis represents the density of observations (all data of all nights and all individuals combined). For details on how the data was collected and the respective units, please refer to the methods section.*

**
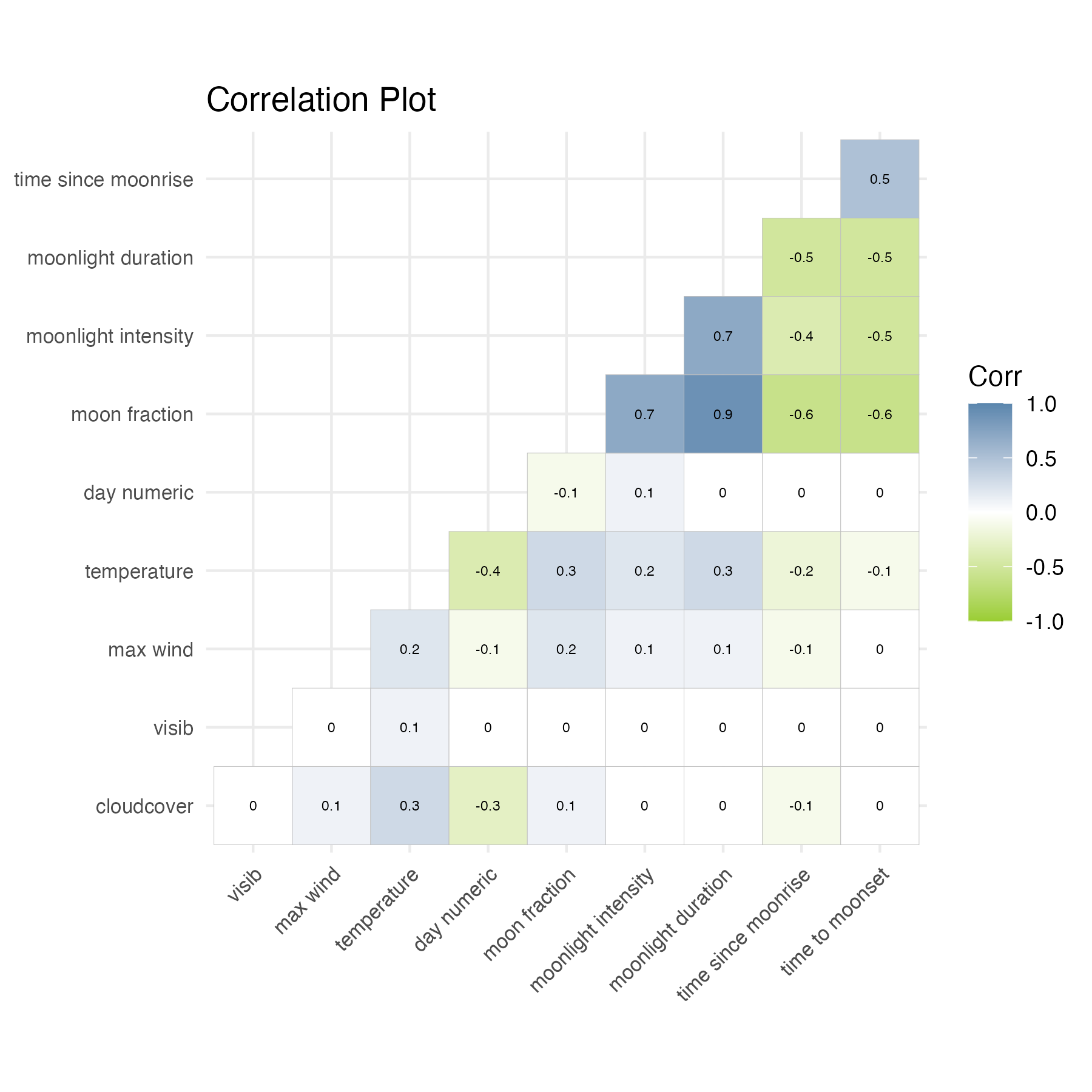
**

**Supplementary Figure S2:** *Correlations between variables used in the models.*


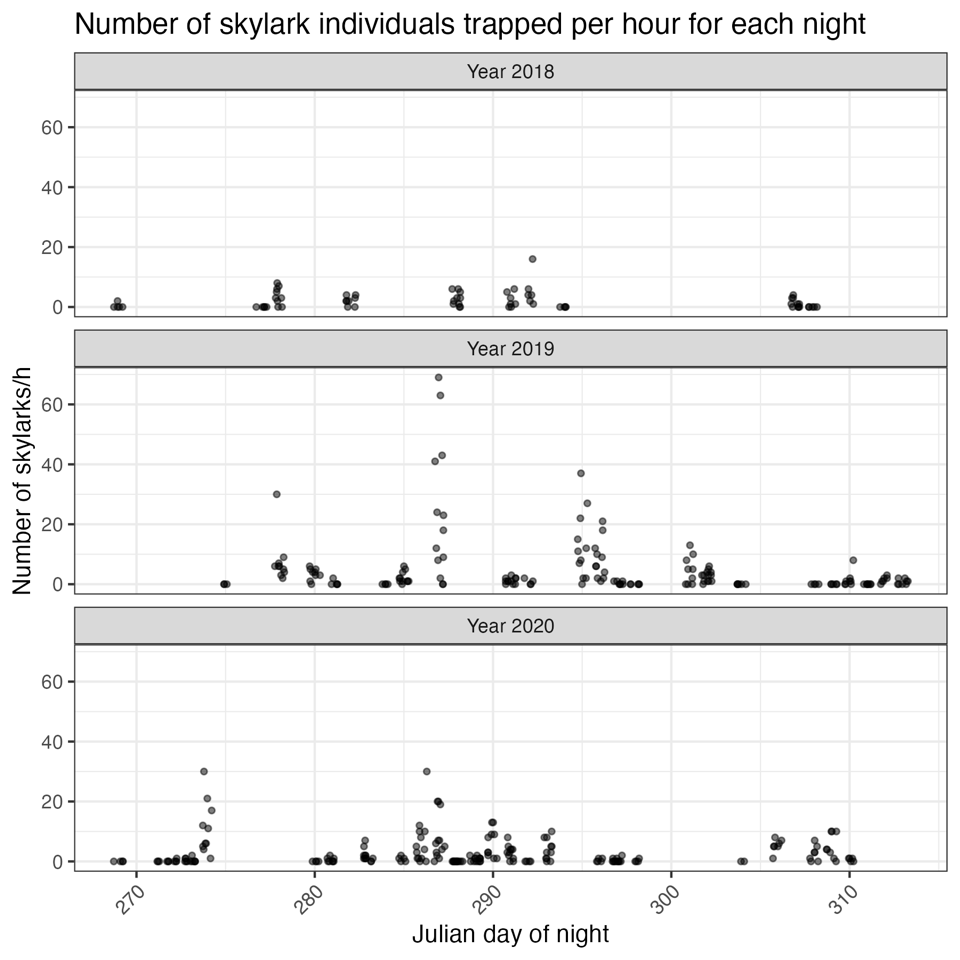


**Supplementary Figure S3:** *Numbers of captured skylarks per hour between 2018 and 2020.*

**Supplementary Table S4:** *Summary of all fixed effects. Given are the respective model, the variable name, as well as the mean, lower and upper CI.*

| **model** | **variable** | **mean** | **lower** | **upper** |
| --- | --- | --- | --- | --- |
| Moon fraction binomial | (Intercept) | -145 | -360 | 58 |
| Moon fraction binomial | moon_fraction | 278 | 27 | 556 |
| Moon fraction binomial | year119 | -17 | -244 | 199 |
| Moon fraction binomial | year120 | -35 | -248 | 171 |
| Moon fraction poisson | (Intercept) | 67 | -1 | 137 |
| Moon fraction poisson | moon_fraction | 70 | -19 | 165 |
| Moon fraction poisson | year119 | 10 | -71 | 83 |
| Moon fraction poisson | year120 | -13 | -90 | 57 |
| Moonlight intensity binomial | (Intercept) | -66 | -229 | 96 |
| Moonlight intensity binomial | illumination | 959 | 369 | 1553 |
| Moonlight intensity binomial | year119 | 35 | -164 | 229 |
| Moonlight intensity binomial | year120 | -9 | -197 | 177 |
| Moonlight intensity poisson | (Intercept) | 104 | 47 | 164 |
| Moonlight intensity poisson | illumination | 310 | 172 | 446 |
| Moonlight intensity poisson | year119 | -7 | -82 | 64 |
| Moonlight intensity poisson | year120 | -36 | -110 | 35 |
| Night moonlight duration binomial | (Intercept) | -189 | -405 | 13 |
| Night moonlight duration binomial | night_moonlight_duration | 27 | 7 | 49 |
| Night moonlight duration binomial | year119 | -13 | -227 | 191 |
| Night moonlight duration binomial | year120 | -41 | -245 | 154 |
| Night moonlight duration poisson | (Intercept) | 67 | -5 | 146 |
| Night moonlight duration poisson | night_moonlight_duration | 5 | -4 | 13 |
| Night moonlight duration poisson | year119 | 16 | -61 | 88 |
| Night moonlight duration poisson | year120 | -12 | -87 | 56 |
| Time since moonrise binomial | (Intercept) | -83 | -191 | 24 |
| Time since moonrise binomial | time_since_moonrise | 10 | 0 | 21 |
| Time since moonrise binomial | year119 | 39 | -63 | 140 |
| Time since moonrise binomial | year120 | 64 | -38 | 167 |
| Time since moonrise poisson | (Intercept) | 138 | 26 | 257 |
| Time since moonrise poisson | time_since_moonrise | -3 | -8 | 2 |
| Time since moonrise poisson | year119 | -7 | -143 | 124 |
| Time since moonrise poisson | year120 | -36 | -171 | 90 |
| Time to moonset binomial | (Intercept) | -63 | -154 | 28 |
| Time to moonset binomial | time_to_moonset | 7 | 0 | 14 |
| Time to moonset binomial | year119 | 43 | -54 | 139 |
| Time to moonset binomial | year120 | 39 | -58 | 136 |
| Time to moonset poisson | (Intercept) | 100 | -15 | 216 |
| Time to moonset poisson | time_to_moonset | 4 | 0 | 9 |
| Time to moonset poisson | year119 | -10 | -148 | 122 |
| Time to moonset poisson | year120 | -52 | -190 | 77 |
